# Substance use and co-occurring mental health conditions among adolescents and young adults in North America: a systematic review

**DOI:** 10.1186/s12888-026-08580-2

**Published:** 2026-09-03

**Authors:** Gemy Kuriakose, Jenny Seo, Negar Asgharipourzahmati, Ashley Lew, Rishika Daswani, Martha J. Ignaszewski, Roberto Sassi, Fiona Choi, Vijay Seethapathy, Reinhard Michael Krausz

**Affiliations:** 1 Faculty of Medical Sciences, Private University in the Principality of Liechtenstein (UFL), Triesen, Principality of Liechtenstein; 2 Department of Parkinson, Sleep and Movement Disorders, Center for Neurology, University Hospital Bonn, University of Bonn, Bonn, Germany; 3 Department of Psychiatry, Institute of Mental Health, University of British Columbia (UBC), Vancouver, BC Canada; 4 Department of Psychiatry, Faculty of Medicine, Mashhad University of Medical Sciences, Mashhad, Iran; 5 Department of Child and Adolescent Psychiatry, BC Children’s Hospital, Vancouver, BC Canada; 6 BC Mental Health and Substance Use Disorders Services, Vancouver, BC Canada

**Keywords:** Substance use, Co-occurring mental health conditions, Adolescents and young adults, North America, Care-related implications

## Abstract

**Background:**

Substance use and co-occurring mental health conditions are common among adolescents and young adults and represent an important clinical and public health concern in North America. Trauma exposure, psychiatric symptoms, and social adversity frequently co-occur with substance-related problems in this population. This systematic review aimed to synthesize evidence on prevalence patterns, associated clinical and psychosocial factors, and care-related implications described in the literature on adolescents and young adults with substance use and co-occurring mental health conditions.

**Methods:**

This systematic review followed PRISMA guidelines and was preregistered in PROSPERO (CRD42024581685). A systematic search was conducted in MEDLINE (Ovid), Embase, PsycINFO, and Google Scholar. Eligible studies examined adolescents and young adults (≤ 25 years) in North America. A combination of subject headings, keywords, and synonyms for the concepts “adolescents,” “young adults,” “substance use disorders,” and “co-occurring mental health conditions” was used. Of 1,882 records identified, 29 studies met the inclusion criteria. Given the heterogeneity of the literature, findings were synthesized narratively.

**Results:**

The included studies showed heterogeneity in population, setting, denominator, and ascertainment method. Across the literature, depressive, anxiety-related, trauma-related, and externalizing mental health presentations were frequently reported alongside substance-related problems in adolescents and young adults. Trauma exposure, adverse childhood experiences, and broader social adversity were also commonly reported in association with substance-related problems across studies. Care-related implications were more limited and were largely derived as future directions mentioned across studies rather than direct evaluations of youth-specific service models.

**Conclusion:**

Co-occurring mental health and substance-related problems were frequently reported among adolescents and young adults in high-risk and service-engaged populations in North America. Trauma and social adversity were prominent across the reviewed literature, while direct evidence for evaluated, integrated, youth-specific care models remains limited. These findings support the relevance of developmentally appropriate, integrated, and trauma-informed approaches to care and highlight the need for further research directly evaluating improved service delivery models for this population.

**Clinical trial number:**

Not applicable.

**Supplementary Information:**

The online version contains supplementary material available at https://doi.org/10.1186/s12888-026-08580-2.

## Background

Substance use and mental health conditions are substantial contributors to the global burden of disease during adolescence [1]. Collectively, these conditions are among the leading causes of disability in young people, and are particularly prominent contributors to the total disease burden in high-income countries [2]. Co-occurring substance-related and mental health conditions are an important clinical and public health concern among transition-aged youth (TAY) [3]. Common mental health presentations that co-occur with substance-related problems include mood and anxiety disorders, personality-related difficulties, trauma-related conditions, and disruptive behavioral disorders [3–5]. Substance-related problems in youth have also been reported alongside bipolar disorder, post-traumatic stress disorder (PTSD), psychotic symptoms, and disruptive behavioral disorders in young people aged 15–24 years [6].

The relationship between substance use and mental health conditions is likely multidirectional, with each condition potentially exacerbating the other. This interaction may contribute to the rising prevalence of both conditions [7]. In the United States, high rates of co-occurrence between mental health disorders and substance use disorders (SUDs) have been reported, particularly among individuals with conduct or antisocial disorders [8]. The risks associated with substance use may have been exacerbated by the global COVID-19 pandemic, resulting in increased overdose mortality and worsening mental health, attributed to factors such as loneliness, economic stressors, and maltreatment [9–11]. These broader social and economic stressors are also reflected in overdose data. In a national chart review of overdose deaths among Canadian youth aged 12–24 in 2016–2017, 94% of deaths occurred among those aged 18–24 ^12^. Among youth aged 20–24, unemployment, collective housing, and being unhoused were overrepresented compared to the general population [12].

There is substantial published evidence of a relationship between traumatic events and adverse childhood experiences (ACEs) and the subsequent use of marijuana, cocaine, prescription drugs, and multiple illicit substances [13, 14]. ACEs have been associated with persistent adverse effects on health and well-being across the life course, including increased morbidity and mortality [15]. The social determinants of health framework provides a useful lens for conceptualizing broader structural and contextual determinants of substance use and related harms, including socioeconomic position, income, employment, discrimination, race/ethnicity, access to healthcare, educational attainment, neighborhood context and housing-related vulnerability [16–21]. The relationship between trauma, mental health conditions, and substance use has been examined in North American studies, particularly among vulnerable youth populations who are homeless or marginalized [22–24].

Addressing adversity, stress-related factors, and substance use in youth is important due to the lasting implications for later health and mental health outcomes [15, 16]. Furthermore, an earlier age of drug initiation has been associated with increased vulnerability to later SUDs [5, 25]. Trauma-related and psychiatric comorbidity may also complicate the clinical course and are important considerations in treatment planning for affected youth and young adults [5, 25].

Although early treatment is important, treatment engagement remains low. National survey data in the United States indicate that many adolescents and young adults with substance use treatment needs do not receive care [26], and evidence from overdose cohorts suggests similarly limited linkage to treatment following non-fatal overdose [27]. Lack of access to appropriate mental health and addiction treatment may be more common among youth affected by adverse social determinants of health, such as low socioeconomic status [16]. There is also a relative lack of youth-specific substance use treatment programs, which may contribute to poorer retention in care and poorer treatment outcomes [28, 29]. Traditional treatment systems often separate psychiatric and addiction services, contributing to fragmented and discontinuous care that may be particularly challenging for young people. The implementation of integrated care frameworks remains inconsistent, and systematic evaluations of their effectiveness are scarce [30]. This lack of evidence underscores the importance of reviews that not only identify associated or contextual domains but also examine existing approaches to inform future service development and policy.

The primary objective of this systematic review was to synthesize evidence on prevalence patterns and associated clinical, psychosocial, and developmental factors related to substance use and co-occurring mental health conditions among adolescents and young adults in North America. Formal mental health diagnoses, symptom-level presentations, and broader psychosocial or developmental correlates were considered separately to avoid treating these domains as conceptually equivalent. The secondary objective was to provide a narrative mapping of care-related implications and emerging service approaches described in the included literature.

## Methods

### Literature search strategy

This systematic review was preregistered in PROSPERO (CRD42024581685) and conducted in accordance with PRISMA guidelines. A systematic search was conducted in MEDLINE, PsycINFO, and EMBASE for relevant studies, utilizing both MeSH and non-MeSH terms customized to the syntax of each database (Appendix A). The search covered the period from January 2014 to September 2024. The primary search was conducted in July 2024 with a subsequent update in September 2024, with the final search completed on September 27, 2024. The search strategy was primarily designed to identify epidemiological studies reporting prevalence patterns and associated or correlational findings in adolescents and young adults with substance-related and co-occurring mental health presentations. As care-related approaches were a secondary objective, relevant service-model literature not indexed using prevalence- or risk-related terminology may have been undercaptured. This was taken into account by presenting the care-related findings as a narrative mapping rather than a comprehensive synthesis of evaluated models of care. Google Scholar was searched using an adapted keyword combination based on the core review concepts (adolescents and young adults, substance use or substance-related problems, and co-occurring mental health conditions) to identify relevant studies in North America. The first 150 results were screened; 142 records with a relevant topic and of an accepted type were retrieved and entered into the review. The same eligibility criteria were applied to the Google Scholar results as to records identified through the database searches. Review articles and studies conducted outside North America were not included. Title and abstract screening was conducted independently by two reviewers, followed by full-text review. Discrepancies were resolved through discussion until consensus was reached.

### Inclusion and exclusion criteria

The present review included English-language publications from 2014 onwards that examined substance use, substance-related problems, or SUDs among adolescents and young adults (aged ≤ 25 years) in North America. Included studies also reported co-occurring mental health diagnoses, symptom-level mental health presentations, or broader psychosocial and developmental correlates. For the purposes of this review, the term ‘co-occurring mental health conditions’ was used as an umbrella descriptor, while formal diagnoses, symptom-level presentations, and broader psychosocial or developmental correlates were distinguished from one another and analyzed separately. Participants up to 25 years old were eligible for inclusion. Wherever possible, the findings were interpreted with particular attention to developmental stage, distinguishing between adolescent, young adult, and mixed youth samples.

### Risk of bias assessment

The risk of bias level of the included studies was appraised using the latest published version of the JBI critical appraisal tools for each study design available at the time of access in 2025 (i.e. cross-sectional, cohort, and case-control) [31–33] The risk of bias for each study was evaluated independently by two reviewers. Any discrepancies were resolved through discussion with a third reviewer. A detailed overview of the risk of bias assessment process and the results are provided in Supplementary Table 1 A-C (Appendix B).

### Data extraction and synthesis

Study selection, screening, and data extraction were managed using the Covidence software. Two reviewers independently extracted data using a pre-piloted extraction form. The extracted variables encompassed study characteristics (country, design, year of publication, sample size, population demographics, setting), diagnostic criteria and measures, prevalence estimates of co-occurring mental health conditions, associated factors, and care-related implications. A third reviewer resolved any discrepancies between the extracted fields by the reviewers. The PRISMA flow diagram (Appendix C) provides a concise overview of the number of records that were identified, screened, assessed for eligibility, and ultimately included in the review. A meta-analysis was not possible due to heterogeneity in the populations, the diagnostic criteria, and the reported outcomes. The synthesis of findings was therefore conducted narratively. Reported correlates and contextual factors were grouped into associated or contextual domains based on thematic similarity across the included studies. Care-related implications and emerging service approaches were synthesized separately as an interpretive map of the literature.

## Results

### Characteristics of included studies

A total of 1,882 articles were identified. After removal of 565 duplicates, 1,317 articles remained. Following title and abstract screening, 393 articles were retained for full-text review, of which 29 met the inclusion criteria for data extraction and final analysis. Of the included studies, 21 were conducted in the United States and 8 in Canada. Of the 29 included studies, 25 used cross-sectional designs, 3 were cohort studies, and 1 was a case-control study. The detailed characteristics of all included studies, including population/setting, age group, index condition or exposure, measurements used, and main evidence contributed, are summarized in Table 1. Additionally, specific details of included studies, such as study aim, reported prevalence rates, and clinical and contextual domains, are available in Supplementary Table 2. Notably, one study was included based on its online availability during the search period, with final journal publication occurring the following year [34].

**Table 1:** Study characteristics and contribution to narrative synthesis

Study	Country	Design	Population / setting	Age group	*N*	Index condition or exposure	Measurements	Main evidence contributed	Domains
Leonard et al., 2023	US	Cross-sectional survey	Youth experiencing homelessness or housing instability receiving community services	Transition-aged youth	100	Methamphetamine use	Validated symptom scales; self-report substance use; ACE exposure measure	Mental health symptoms among methamphetamine-using unstably housed youth	C; T; E
Mann et al., 2014	US	Cross-sectional study	Adolescents assessed in an eating disorders program	Adolescents	290	Eating disorders and substance use	Diagnostic interview; eating disorder assessment; depression symptom scale	Substance use among adolescents with eating disorders	C; D
Rhew et al., 2017	US	Cohort study	Community-based school cohort	Adolescents	521	Early adolescent depression	DSM-based diagnostic interviews	Longitudinal association between early depression and later AUD/CUD	C
Adams et al., 2015	US	Cross-sectional study	Disaster-exposed adolescents	Adolescents	2,000	PTSD, MDE, and SUD following tornado exposure	Structured diagnostic interview; substance use screening tool	Co-occurring PTSD, MDE, and SUD in trauma-exposed youth	C; D; T; E
Goodwin et al., 2014	US	Cross-sectional survey	College students	Young adults	1,799	Hookah use	Survey-based self-report	Mental health and substance use correlates of hookah use	C
Sultan et al., 2025	US	Cross-sectional study	National adolescent survey sample	Adolescents	10,123	Cannabis use/CUD among adolescents with MDD or BD	DSM-based structured diagnostic interview	Cannabis use and CUD among adolescents with mood disorders	C; D; T
Freibott et al., 2024	US	Cross-sectional study	College students from the Healthy Minds Study	Young adults	176,191	Opioid misuse	Validated symptom scales; self-reported diagnosis and substance use	Opioid misuse and co-occurring anxiety/depression symptoms or diagnoses	C
Single et al., 2024	US	Cross-sectional epidemiological study	Nationally representative emerging adult sample	Young adults	5,194	Cannabis use and social anxiety disorder	DSM-based structured diagnostic interview	Cannabis use in relation to social anxiety and other psychiatric disorders	C; D
Xu et al., 2024	US	Cross-sectional analysis	Adults from NSDUH, including young adults	Young adults	168,859	Adolescent substance use and adult MDD	DSM-based survey items	Association between SUD and MDD, with demographic and socioeconomic correlates	C; D; E
McKnight et al., 2023	US	Cross-sectional study	Youth in OUD and eating disorder programs	Adolescents and young adults	90	OUD, eating disorders, ACEs, and resilience	Formal clinical diagnosis; ACE/resilience measures	ACE burden and resilience factors among youth with OUD and eating disorders	C; T; S
Striley et al., 2022	US	Cross-sectional analysis	Students from the Healthy Minds Study	Young adults	47,016	E-cigarette use and non-suicidal self-injury	Survey-based self-report	Substance use patterns among students with non-suicidal self-injury	C; D
Salmon et al., 2023	Canada	Cross-sectional epidemiological study	Adolescents from the Ontario Child Health Study	Adolescents	2,910	Child maltreatment and peer victimization	Survey-based self-report	Child maltreatment, peer victimization, and adolescent substance use	T; S
Krebs et al., 2021	Canada	Cohort study	Youth diagnosed with OUD in British Columbia acute care settings	Adolescents and young adults	13,009	OUD diagnosis and co-occurring mental health disorders	Administrative health data; ICD codes; OAT medication records	Mental health disorders among adolescents and young adults with OUD	C
Dawson-Rose et al., 2020	US	Cross-sectional study	Homeless or unstably housed transition-aged youth recruited from community organizations	Transition-aged youth	100	Trauma, mental health symptoms, and substance use	Validated symptom scales; brief substance use screening	Trauma and mental health symptoms among unstably housed youth who use substances	C; T; E
Regan & Tubman, 2020	US	Cross-sectional study	Adolescents receiving outpatient substance use treatment	Adolescents	394	ADHD subtypes among youth in substance use treatment	DSM-based structured diagnostic interview	ADHD subtypes and co-occurring psychiatric symptoms among adolescents in substance use treatment	C; D
Cheung et al., 2019	Canada	Cross-sectional study	Students from the Ontario Student Drug Use and Health Survey	Adolescents	2,945	Co-occurring substance use problems and internalizing symptoms	Validated distress scale; substance use screening tools; self-report items	Prevalence of co-occurring substance use problems and internalizing symptoms	C
Ramos-Olazagasti et al., 2017	US	Cohort study	Puerto Rican youth cohort	Adolescents	1,259	Childhood adversity and early alcohol initiation	DSM-based alcohol module; self-reported alcohol initiation	Childhood adversity and early alcohol use initiation	T; S
Masroor et al., 2019	US	Cross-sectional study	Adolescent psychiatric inpatients	Adolescents	800,614	Conduct disorder and SUD-related hospitalization	Administrative inpatient data; ICD-9 codes	Substance use disorders among adolescents hospitalized with conduct disorder	C; D
Conway et al., 2016	US	Cross-sectional study	National adolescent survey sample	Adolescents	10,123	Prior mental disorders and substance use transitions	DSM-based structured diagnostic interview	Mental disorders associated with transitions across stages of alcohol and drug use	C
Edlund et al., 2015	US	Cross-sectional study	Adolescents from NSDUH	Adolescents	112,600	Major depressive episode and nonmedical prescription opioid use	DSM-based survey assessment; self-report opioid misuse	Association between MDE, nonmedical prescription opioid use, and opioid abuse/dependence	C; D; S
Desai et al., 2022	US	Cross-sectional analysis	High-school students from YRBSS	Adolescents	125,550	Hallucinogen use, hopelessness, suicidality, and substance co-use	Survey-based self-report	Mental health and polysubstance use correlates among adolescents reporting hallucinogen use	C
Carliner et al., 2017	US	Cross-sectional study	National adolescent survey sample	Adolescents	6,379	Potentially traumatic events, externalizing disorders, and SUD	DSM-based diagnostic interview; trauma exposure assessment	Trauma exposure patterns among adolescents with SUD and externalizing disorders	C; T
Brinkman et al., 2015	US	Cross-sectional study	Adolescents from NHANES	Adolescents	2,517	ADHD, conduct disorder, tobacco use, and alcohol use	DSM-based diagnostic interview; self-report substance use	Tobacco and alcohol use among adolescents with ADHD and/or conduct disorder	C
Henderson et al., 2017	Canada	Cross-sectional study	Youth seeking services across participating agencies	Adolescents and young adults	2,576	NEET status, substance use, and mental health problems	Self-report screening tool for probable service need	Co-occurring internalizing, externalizing, and SUD-related problems among NEET and non-NEET youth	C; S; E
Hawke et al., 2018	Canada	Cross-sectional study	Youth receiving addiction and concurrent disorder services	Adolescents and young adults	1,232	Cannabis/substance use and co-occurring mental health concerns	Substance use assessment; GAIN-SS screening tool	Co-occurring SUD and internalizing/externalizing problems by age and sex	C; D
Brownlie et al., 2019	Canada	Cross-sectional study	Grade 7 and 8 students in Ontario	Adolescents	1,360	Concurrent mental health and substance use problems	GAIN-SS; adapted student drug use and health survey items	Prevalence of concurrent mental health and substance use problems in early adolescence	C
Marshall et al., 2023	Canada	Case-control study	Alberta youth with continuous provincial health insurance coverage	Adolescents and young adults	9,240	Pre-existing mental health disorders and OUD	Administrative health data; ICD-based diagnoses; opioid dependence program records	Pre-existing anxiety, depressive, and alcohol-related disorders among youth with OUD	C
Ware et al., 2024	US	Cross-sectional study	Youth receiving treatment in community mental health centres	Adolescents and young adults	2,252,281	Primary anxiety, depression, or ADHD diagnosis with co-occurring high-risk SU/SUD	Administrative treatment records	High-risk substance use or SUD among youth with primary anxiety, depression, or ADHD diagnoses	C; D
Aderibigbe et al., 2022	Canada	Cross-sectional study	Youth assessed in community or residential mental health agencies in Ontario	Adolescents	48,118	Individual, family, and clinical factors associated with substance use	interRAI Child and Youth Mental Health Assessment	Clinical, family, trauma, and educational correlates of substance use among youth in mental health services	C; D; T; S; E

Abbreviations for Contextual Domains Column: C = clinical; D = demographic; E = environmental; S = social; T = trauma-related

Other Abbreviations: ACE = adverse childhood experience; ADHD = attention-deficit/hyperactivity disorder; AUD = alcohol use disorder; BD = bipolar disorder; CUD = cannabis use disorder; GAIN-SS = Global Appraisal of Individual Needs–Short Screener; MDD = major depressive disorder; MDE = major depressive episode; NEET = not in education, employment, or training; NSDUH = National Survey on Drug Use and Health; OAT = opioid agonist treatment; OUD = opioid use disorder; SUD = substance use disorder; TAY = transition-aged youth; YRBSS = Youth Risk Behavior Surveillance System

Note: Because included studies varied substantially in population, outcome definition, and assessment method, findings are presented descriptively and not intended as directly comparable prevalence estimates

As previously noted, this review selectively targeted evidence on adolescents and young adults. Specifically, sixteen studies involved adolescents, two studies included transition-aged youth, five studies examined young adults, and six studies had both adolescents and young adults. Eleven studies had a sample size of more than 10,000 participants, twelve studies included participants between 1,000 and 9,999, and six studies had a sample size below 1,000 participants.

Most included studies used survey-based assessments or structured interviews to assess the presence and severity of mental health or SUDs. DSM-based questions or diagnostic interviews were implemented in 17 studies. Although many of these findings were based on self-reports, these assessments were reported to have high reliability and validity that align with DSM diagnoses. Other surveys assessed the probable need for care services by measuring self-reported symptoms, rather than assessing based on diagnostic criteria. Five studies reported formal diagnoses of mental health or SUDs made by clinicians. The diagnoses were based on the DSM or ICD-9/ICD-10 codes in health administrative databases. The specific assessments used for each study are summarized in Table 1.

### Risk of bias assessment

The risk of bias of all 29 included studies was assessed to inform the interpretation of review findings. Risk-of-bias judgements were based on whether predefined critical methodological domains were met, rather than on the calculation of an overall numerical cutoff score. The results of the risk of bias assessment and the identified critical methodological domains for each study type are specified in Supplementary Tables 1 A-C. Responses of both ‘No’ and ‘Unclear’ within these critical domains were considered to increase the risk of bias. Studies were classified as having a low risk of bias if they successfully met all critical domains, moderate risk of bias if one critical domain or multiple minor areas were missed, and high risk of bias if they failed to meet two or more critical domains. Overall, the included studies were judged to have a low-to-moderate risk of bias. Most cross-sectional studies were characterized by the presence of clearly defined inclusion criteria, detailed descriptions of study populations, and the application of valid measures for exposures and outcomes. The main limitations were related to the identification and management of confounding factors, which were unclear in two studies and not accounted for in one study [35–37]. The three cohort studies generally met several key methodological domains, including adequate follow-up and appropriate statistical analysis. However, the appraisal was not uniformly without limitations, as it was unclear in two studies whether participants were free of the outcome at baseline [14, 38]. These unclear ratings were considered in the overall judgement and support a more cautious interpretation of the cohort evidence. The single case-control study met all methodological domains, with clear comparability between groups and appropriate statistical methods [39].

### Associated or contextual domains

Five major associated or contextual domains were identified: clinical, demographic, trauma-related, social, and environmental. These domains were recurrently described in relation to adolescent substance use and co-occurring mental health conditions across the included studies. However, these domains are not conceptually equivalent to formal psychiatric comorbidity. Rather, they reflect a broader range of correlates, vulnerabilities, and contextual factors described in the included literature. For the purposes of this review, the clinical domain refers to mental health presentations and clinically relevant characteristics reported alongside substance-related problems, rather than formally diagnosed psychiatric comorbidity alone. The demographic domain refers to characteristics such as age, sex or gender, and race or ethnicity. The trauma-related domain refers to trauma exposure, ACEs, and related histories of violence or abuse. The social domain refers to interpersonal and relational influences, including family functioning, peer relationships, and social marginalization. The environmental domain refers to broader contextual influences such as socioeconomic disadvantage, housing instability, and not in education, employment, or training (NEET) status.

#### Clinical domain

The clinical domain was the most frequently reported, with relevant factors described in 27 studies. The findings from these studies recurrently suggested associations with substance use and related disorders across study contexts. For example, one study found that cannabis use was more prevalent among adolescents with major depressive disorder (MDD) than among controls without mood disorders [34]. Specifically, 32.9% of individuals diagnosed with major depressive disorder reported cannabis use, in contrast to 16.9% among the control group without mood disorders. Cannabis use in a population of grade 7 and 8 students also showed 10-times the odds of having above-threshold externalizing and internalizing problems [40]. Demonstrating the prevalence of these clinical overlaps, Cheung et al. found that 12.0% of their adolescent sample reported both internalizing symptoms and substance use problems, a co-occurrence that escalated significantly from 7.5% in grade 9 to 14.2% in grade 12 [41]. Hallucinogens use was also associated with feeling sad and hopeless, as well as considering and planning suicide [42]. Furthermore, prior mental health disorders were associated with increased risk of transitioning across stages of alcohol and drug use, from nonuse to initiation, and then to problematic use [43]. Additionally, neurodevelopmental conditions were addressed in several studies. Regan & Tubman (2020) reported that adolescents receiving substance use treatment showed high rates of attention-deficit/hyperactivity disorder (ADHD), which were associated with substance-related problems regardless of subtype [44]. In line with this evidence, a cross-sectional study with adolescents also showed that ADHD and Conduct Disorder were associated with an increased likelihood of substance use [45]. Stress-related conditions, including PTSD, were also frequently described in relation to substance-related problems. In one study, 96.4% of methamphetamine-using youth had symptoms consistent with PTSD, compared with 72.7% of youth who had not used methamphetamine [46]. Trauma history and ACEs were also reported across studies as relevant clinical correlates, consistent with prior literature linking early adversity to later health outcomes [47]. McKnight et al. (2023) reported that high ACE exposure was common among youth with opioid use disorder (OUD) and feeding and eating disorders (FEDs), with more than half reporting four or more ACEs [48]. Highlighting the broader psychiatric burden within this specific population, Krebs et al. (2021) found that 79.2% of adolescents and 68.0% of young adults diagnosed with OUD had a concurrent mental health disorder [49]. Similarly, Dawson-Rose et al. (2020) found that 77% of homeless youth reported four or more ACEs, which often co-occurred with substance use and high rates of PTSD, depression, and anxiety [35]. Overall, these findings suggest that internalizing, neurodevelopmental, and trauma-related conditions were frequently reported alongside substance-related problems in adolescents and young adults.

#### Demographic domain

The demographic domain was identified in 12 studies, with associations varying in strength depending on the population and context. Xu et al. (2024) found that young adults between the ages of 18 and 25 years had the highest prevalence of MDD, and that past-year SUDs were associated with increased MDD prevalence overall [50]. Findings regarding gender differences were mixed. Striley et al. (2022) found that students with past-year non-suicidal self-injury were more likely to be female, and that substance use was common in this group, with 61.5% reporting alcohol use and 37.6% reporting marijuana use [51]. Edlund et al. (2015) similarly reported that female adolescents had a greater likelihood of nonmedical prescription opioid use [52]. In contrast, other studies reported that male adolescents had higher rates of substance use or certain mental health conditions. For example, one study reported higher rates of regular cannabis use among male adolescents with eating disorders compared to females [53]. Racial and ethnic differences were also described across studies. Single et al. (2024) found that being White was associated with increased odds of co-occurring cannabis use and social anxiety disorder in emerging adulthood, whereas Adams et al. (2015) identified race as a demographic factor examined in relation to post-disaster comorbidity profiles [54, 55]. Taken together, these findings suggest that demographic patterns varied across study contexts and may be relevant when interpreting co-occurring substance-related and mental health presentations in youth.

#### Trauma-related and social domain

The trauma-related and social domain was described in a total of 15 studies, with 9 and 6 studies respectively. Exposure to interpersonal trauma was found to be associated with substance use and mental health conditions. Carliner et al. (2017) reported that SUDs were more common among adolescents exposed to interpersonal trauma (31.2%) than among those without such exposure [56]. Adams et al. (2015) found that adolescents affected by tornadoes in 2011 experienced comorbid PTSD, major depressive episodes, and substance use, with prior traumatic events and loss of services serving as significant risk factors for these comorbidity patterns [54]. Ramos-Olazagasti et al. (2017) found that cumulative childhood adversities, such as physical and emotional abuse, parental antisocial behavior, and exposure to violence, were associated with early alcohol use, particularly among young girls [14]. These factors frequently intersected, reflecting the complex interplay between individual experiences and broader social environments. Further illustrating this intersection, Henderson et al. (2017) reported that youth experiencing social marginalization through disengagement from employment, education, or training were significantly more likely to endorse having disturbing memories compared to their engaged peers [37]. Social determinants such as lack of supportive relationships, peer substance use, and social marginalization were commonly associated with substance-related problems and co-occurring mental health presentations, suggesting the potential relevance of protective social supports, networks, and resilience-related factors. The findings on social factors also suggest that family functioning and school connectedness may be relevant in relation to public health interventions. This is consistent with evidence that positive childhood experiences, such as supportive parent-child relationships and school engagement, are associated with lower risk of adverse mental and physical outcomes [57]. These protective factors may warrant further consideration in youth-focused prevention approaches.

#### Environmental domain

The environmental domain was described in 6 studies. Across these studies, factors such as peer substance use, family environment, NEET status, and socioeconomic disadvantage were recurrently reported. Striley et al. (2022) reported a high prevalence of alcohol and marijuana use among students with past-year non-suicidal self-injury (61.5% and 37.6%, respectively) [51]. Peer substance use was identified as another social factor in other studies and may contribute to these patterns [6]. Another consistent factor was the family environment. Aderibigbe et al. (2022) reported that parental substance use and lack of family support were associated with adolescent substance use, alongside disrupted education [6]. These factors commonly co-occur as ACEs, and their effects are often difficult to disentangle due to shared underlying adversity. Henderson et al. (2017) found that youth with NEET status were more likely to screen positive for concurrent internalizing, externalizing and problematic substance use concerns compared to non-NEET peers [58]. Furthermore, the structural impact of NEET status was reflected in their housing environments, as these youth were significantly more likely to experience high-risk housing situations and to present for care through the housing and support sector [37]. Socioeconomic disadvantage, such as low household income, when combined with early substance use, was associated with a higher risk for MDD in another study [50]. Homelessness was also identified as a severe environmental vulnerability, with Dawson-Rose et al. (2020) reporting that 50% of the transitional age youth in their study were experiencing homelessness alongside high rates of trauma and substance use [35]. These factors reflect broader systemic and contextual influences on substance use and suggest the importance of considering youth substance use within its broader social and economic context. Approaches that address structural determinants, such as poverty, housing instability, and substance availability, may therefore be relevant alongside clinical and individual-level strategies.

Overall, the presence of these vulnerabilities suggests the relevance of integrated approaches that address clinical, demographic, trauma-related, social, and environmental factors associated with substance-related and mental health problems in adolescents and young adults.

### Prevalence patterns across study contexts

In addition to the associated or contextual domains identified across studies, the included literature also reported prevalence estimates of co-occurring SUDs and mental health conditions among adolescents and young adults. Given the substantial differences in population, denominator, setting, and ascertainment method across the included studies, prevalence findings were synthesized according to index condition and study context. This approach was taken to avoid interpreting the findings as reflecting a single pooled prevalence construct.

#### Mental health conditions in substance-related populations

Several studies reported mental health conditions within populations associated with substance use, though the reported estimates showed substantial variability. Major depressive episodes or depressive symptoms were examined in 16 studies, anxiety-related conditions in 12 studies, and PTSD or trauma-related disorders in 4 studies. In a Canadian case-control study of young people with OUD, anxiety disorders were reported in 72.7% of cases, depressive disorders in 34.7%, and co-occurring anxiety and depressive disorders in 31.8% [39]. Other studies reported lower estimates, including approximately 30–33% for some co-occurring conditions or symptom clusters, illustrating the heterogeneity in study populations, definitions, and measurement approaches [14, 56]. Taken together, these findings indicate that mental health symptoms and diagnoses were frequently reported among populations of youth who use substances, while underscoring the importance of distinguishing between diagnostic, screening-based, and self-reported estimates.

#### Substance-related outcomes in psychiatric populations

Substance-related outcomes were also reported in populations defined by psychiatric or behavioral presentations. Externalizing and neurodevelopmental disorders, including ADHD, conduct disorder, oppositional defiant disorder, and antisocial traits, were described in 3 studies. Reported estimates varied substantially across studies, ranging from 5.2% for co-occurring high-risk substance use or SUD among youths with ADHD as a primary diagnosis in community mental health settings to 76.1% for past-year drug abuse in adolescents receiving outpatient substance abuse treatment, likely reflecting differences in study population and outcome definition [44, 59]. Eating disorder populations were also represented, with Mann et al. (2014) reporting lifetime prevalence of any substance use of 24.6% in anorexia, 48.7% in bulimia nervosa, and 28.6% in eating disorder not otherwise specified (EDNOS) [53]. These findings suggest that substance-related problems were reported across a range of psychiatric and behavioral populations. However, direct comparison between studies was limited by heterogeneous denominators and case definitions.

#### Co-occurring presentations in service-engaged and administrative samples

Several studies described co-occurring presentations in service-engaged, clinically selected, or administratively defined samples. These studies frequently employed diagnostic codes, service records, or treatment-based populations as denominators, resulting in analytically distinct prevalence estimates compared to those reported in symptom-based or population-derived studies. In addition, 8 studies included in this review did not report prevalence estimates for co-occurring conditions, underscoring significant limitations in the available evidence base and variability in how co-occurrence was defined and measured.

#### Trauma and adversity patterns relevant to co-occurrence

Trauma exposure and ACEs were examined in 4 studies in relation to substance-related outcomes. Reported estimates varied considerably by population and definition. These estimates ranged from a 33.1% cumulative probability of alcohol initiation by age 14 in a Puerto Rican youth sample to 80% of adolescents and young adults with OUD reporting four or more ACEs, compared with 34.0% of those with feeding and eating disorders [14, 48]. Carliner et al. (2017) reported that SUDs were more common among adolescents exposed to interpersonal trauma (31.2%) [56], and McKnight et al. (2023) found that high ACE scores were common among adolescents and young adults with OUD [48]. Overall, these findings suggest that trauma and adversity are prominent contextual features in youth substance-related populations. However, the included studies do not consistently establish temporality or causality.

The preceding sections synthesize the primary evidence on prevalence patterns and associated domains reported across the included studies. The following section is more interpretive in nature and provides a narrative mapping of care-related implications and emerging service approaches described in the literature.

### Care-related implications and emerging service approaches

The literature included in this review provided limited direct evidence on evaluated models of care for adolescents and young adults with SUDs and co-occurring mental health conditions. Many of the studies were observational, descriptive, or epidemiological and did not directly assess treatment models, integrated service structures, or intervention effectiveness. Therefore, this section presents a narrative mapping of care-related implications, emerging or potential future service approaches, and related recommendations described or suggested in the literature, rather than a synthesis of evidence-based, effective models of care.

The identified care-related implications, emerging service approaches, and related recommendations are summarized in Table 2.

**Table 2:** Potential service approaches for future research and development mentioned across the included studies

Category	Potential service approaches	Studies
Prevention and Early Identification	Screening for early identification • Routine screening for early detection and intervention (e.g. stress, depression, PTSD, SUD screening in schools, shelters) • Targeted screening and outreach for higher-risk groups (e.g. prior trauma, experience of disaster, early depression, low-income, parental conflict) • Screening for co-occurring mental health conditions (e.g. trauma history in youth presenting with EDs) • Post-traumatic event screening (e.g. within 3–6 months) Programs and education • Targeted programs intended to reduce the risk of CUD/AUD/SUD or co-use (e.g. for individuals with or at risk of depression, to reduce cannabis misuse) • Education on risk and harms, especially for higher-risk groups • Education on skills (e.g. coping skills, positive parenting techniques) • Recognizing conditions such as substance use and conduct problems as potential indicators of trauma and ongoing violence	Rhew et al., 2017 Adams et al., 2015 Goodwin et al., 2014 Sultan et al., 2025 Xu et al., 2024 Salmon et al., 2023 Masroor et al., 2019 Carliner et al., 2017
Assessment and Risk Stratification	Screening to identify higher-risk individuals • Screening for co-occurring conditions (e.g. MDD and PTSD in populations using methamphetamine, substance-using youth with eating disorders, SUD screening when PTSD/MDE is detected) • Screening for concurrent substance use (e.g. smoking, alcohol, cannabis) Interventions • Stratified intervention approaches according to level of risk (e.g. use vs. use disorder) • Assessment and treatment of co-occurring conditions with shared psychopathology (e.g. GAD, MDD)	Leonard et al., 2023 Mann et al., 2014 Adams et al., 2015 Goodwin et al., 2014 Sultan et al., 2025 Xu et al., 2024 Single et al., 2024
Integrated Care and Psychosocial Treatment	Treatment • Integrated approaches addressing both mental health and substance-related problems (e.g. CBT, TF-CBT, motivational interviewing) • Assessment and treatment of co-occurring conditions • Interventions specific to mental health or substance use disorder (e.g. depression, as it may reduce CUD treatment efficacy), with attention to resilience and well-being • Trauma-focused and trauma-informed care (e.g. for history of abuse or neglect) • Family-centered care (e.g. addressing caregiver distress) • Gender-specific support (e.g. girls, due to higher depression risk) Education • Group psychoeducation (e.g. trauma processing in community settings) Psychosocial • Addressing social factors (e.g. family, school) • Holistic approaches to risk reduction/assessment, recovery, and social factors (e.g. family, school) • Interventions addressing interpersonal violence (e.g. youth with CD and ODD) Settings • Interventions delivered in school clinics • Peer groups and navigators intended to improve access	Rhew et al., 2017 Adams et al., 2015 Sultan et al., 2025 Freibott et al., 2024 Single et al., 2024 Xu et al., 2024 Salmon et al., 2023 Carliner et al., 2017 Hawke et al., 2018 Ware et al., 2024 Aderibigbe et al., 2022
Technology and Innovation	Digital tools • App- or online-based approaches for subthreshold symptoms (e.g. CBT-based) • Brief tools intended to support efficient clinical use (e.g. single-item screens)	Adams et al., 2015 Single et al., 2024
Policy and System-Level Interventions	Development • Community partnerships intended to address access barriers (e.g. schools, shelters, and telehealth) • One-stop-shop services and integrated service hub models • Need for further research to develop and evaluate youth-specific treatments Policy considerations • Policy considerations related to reducing access (e.g. taxes, age limits) • Policy considerations related to youth access to high-THC products and insurance coverage for integrated care Education • Supportive education • Peer-support and peer-education elements intended to facilitate help-seeking • Supportive education intended to reduce stigma and improve linkage to resources • Training or continuing education for healthcare professionals in SUD treatment as potentially relevant service components	Adams et al., 2015 Goodwin et al., 2014 Sultan et al., 2025 Freibott et al., 2024 Henderson et al., 2017 Hawke et al., 2018 Ware et al., 2024 Aderibigbe et al., 2022
Long-Term Monitoring and Follow-up	• Longer-term monitoring across adolescence • Follow-up of co-occurring conditions into adulthood	Rhew et al., 2017 Xu et al., 2024

This table is meant to be a thematic map, as the following categories and service approaches were not directly evaluated in the literature. AUD: alcohol use disorder; CBT: Cognitive Behavioral Therapy; CD: conduct disorder; CUD: cannabis use disorder; ED: eating disorder; GAD: generalized anxiety disorder; MDD: major depressive disorder; MDE: major depressive episode; ODD: oppositional defiant disorder; PTSD: post-traumatic stress disorder; SUD: substance use disorder; TF-CBT: Trauma-Focused Cognitive Behavioral Therapy; N/A: not available

#### Prevention and early detection

Prevention and early identification strategies were described in 8 studies (see Table 2). These reports included routine and targeted screening approaches, school-based prevention components, and outreach strategies for youth considered to be at elevated risk of substance-related and co-occurring mental health conditions. Educational settings were also discussed, including the potential role of school-based intervention programs in addressing alcohol, cannabis, tobacco, and related mental health problems among adolescents and young adults [7, 38, 60]. Prevention-oriented components suggested in the literature included early identification, family- and parenting-focused preventive strategies, and targeted attention to higher-risk groups [61]. At the same time, the included studies did not consistently report whether these approaches incorporated skills-based components or other elements necessary to evaluate their likely effectiveness in changing youth substance use behavior. Some studies further suggested that SUDs and conduct-related problems may co-occur with trauma exposure or violence-related adversity, supporting consideration of comprehensive assessment and early identification approaches in vulnerable youth populations [34].

#### Assessment and risk stratification

Assessment and stratification-related elements were described in 7 studies (see Table 2). These reports emphasized screening, clinical assessment, and monitoring approaches for youth considered to be at elevated risk, including those with co-occurring substance use and mental health conditions like MDD or PTSD [34, 50, 54]. Some studies suggest that identifying concurrent use of substances, such as cigarettes and cannabis, may be relevant when examining broader patterns of co-use or polysubstance involvement [60]. The literature also suggests that assessing co-occurring conditions, such as MDD and anxiety disorders, may be relevant given their frequent overlap with certain substance-related problems [55]. However, these studies did not evaluate formal risk stratification frameworks or compare specific stratified care pathways. Rather, they collectively suggest that structured assessment of substance use severity, co-occurring mental health presentations, and broader psychosocial vulnerability may be relevant to service planning in youth populations.

#### Integrated care and psychosocial treatment

Integrated care and psychosocial treatment-related elements were discussed in 11 studies. Across the included literature, the need for approaches that address both substance-related problems and co-occurring mental health conditions was described recurrently. Several studies mention psychosocial or supportive components such as cognitive behavioral therapy (including trauma-focused cognitive behavioral therapy), motivational interviewing, family-centered approaches, psychoeducation, and peer- or school-linked support components [34, 50, 54, 62, 63]. However, these elements were not consistently evaluated within integrated service models and the extent to which they were implemented as coordinated care pathways varied across studies. The literature also suggested that social and interpersonal factors, including family functioning, caregiver distress, school-related difficulties, and exposure to violence or neglect, may be relevant when considering psychosocial support needs in this population [6, 54, 56, 63]. Taken together, these studies support the view that integrated and trauma-informed psychosocial approaches are clinically relevant for youth with substance-related and co-occurring mental health problems, while also highlighting the limited direct evidence for evaluated, youth-specific integrated treatment models.

#### Technology and innovation

Technology- and innovation-related approaches were discussed in 2 studies. These studies referred to brief assessment-oriented approaches, including app- or online-based tools for subthreshold symptoms, as well as single-item and routine screening strategies intended to support the efficient identification of co-occurring mental health and substance use problems in young people across care settings [54, 55]. Overall, the evidence base in this area was limited, and the approaches were described primarily as complementary or emerging components rather than as evaluated, stand-alone care models.

#### Policy and system-level interventions

Policy and system-level interventions were discussed in 8 studies. Across the included literature, these elements were described primarily as service implications, structural recommendations, and proposed approaches to reduce barriers to care, rather than as evaluated models of service delivery. Reported elements included schools, shelters, and telehealth-based supports as approaches intended to improve access to care [54]. Integrated, youth-oriented service hub models were also described as approaches intended to improve access to coordinated and developmentally appropriate care, although further research is needed to refine and evaluate youth-specific treatment effectiveness in this area [36]. Several studies referred to broader policy and care related considerations, including concerns around youth access to substance-use products and the need for prevention and treatment strategies addressing cannabis use and cannabis use disorder in clinically vulnerable adolescents [34, 60]. Some studies described supportive elements such as youth-oriented education, informal help-seeking pathways, and peer-oriented support or training to facilitate help-seeking, although these were not presented as comprehensive models of care [6, 62]. Other reports highlighted training and education in SUDs and co-occurring mental health conditions for healthcare professionals as potentially relevant components of care [59].

#### Long-term monitoring and follow-up

A small proportion of studies highlighted the importance of longer-term monitoring and follow-up, particularly given that substance-related and co-occurring mental health conditions may extend from adolescence into adulthood. Two studies suggested that longitudinal monitoring of early substance use and depressive symptoms is important for understanding later outcomes, as long-term follow-ups are needed to confirm the causal risk trajectories between adolescent substance use and adult depression [38, 50]. Tracking these co-occurring issues from adolescence into adulthood can help clarify risk patterns and inform prevention and treatment planning over time.

## Discussion

Reported estimates of co-occurring mental health and substance use conditions varied substantially across the included studies, ranging from low rates in some narrowly defined diagnostic contexts to higher rates in general populations, such as among college students. This heterogeneity likely reflects differences in study populations, assessment methods, case definitions, and sampling strategies. Across the reviewed literature, depression, anxiety, trauma exposure, and ACEs were recurrently reported alongside substance-related problems in adolescents and young adults. The differing developmental stages of study populations may also have contributed further to this heterogeneity, with some studies including only adolescents or young adults, and others using mixed youth samples. Overall, the findings suggest a substantial degree of co-occurrence between substance-related problems and internalizing, externalizing, and trauma-related mental health presentations in youth.

The reviewed literature also points to important gaps in service provision for adolescents and young adults with substance-related and co-occurring mental health conditions. Most studies provided observational evidence or suggestions for future models of care rather than direct evaluations of youth-specific service models. Across the included studies, suggestions for care-related elements most commonly involved family engagement, psychosocial support, trauma-informed approaches, and screening or assessment strategies adapted to youth vulnerability and service context. Emerging approaches such as early psychosis programs, trauma-informed care, and family-centered interventions illustrate the potential relevance of coordinated and developmentally appropriate services for improving engagement and retention [54, 64]. Some reports also referred to interventions more commonly established in adult populations, such as OAT and neurocognitive rehabilitation following non-fatal overdose. However, within North America, early intervention and integrated youth-oriented approaches are described inconsistently, and evidence and evaluation of these care pathways remains limited. Given that many adolescents and young adults do not consistently receive timely access to evidence-based addiction treatment following acute care presentations, our findings highlight the relevance of developmentally appropriate, integrated, and trauma-informed approaches for youth with substance-related and co-occurring mental health conditions, while also highlighting the limited direct evidence for evaluated youth-specific care models [27, 30]. In addition, pharmacological approaches with established efficacy in adults appear to remain understudied and under-evaluated in youth populations. Taken together, the findings underscore the need for further research evaluating developmentally appropriate, integrated, and trauma-informed care models for this population.

### Strengths and limitations

Because this review was restricted to North American studies, the findings should be interpreted primarily in relation to the service structures and clinical contexts of the United States and Canada rather than being generalized globally. This review has several strengths, including PROSPERO preregistration, PRISMA-guided methodology, systematic searching across multiple databases, supplementary grey literature searching, and independent dual screening with consensus-based resolution of discrepancies. The review also sought to move beyond a simple prevalence summary by considering associated clinical, psychosocial, and developmental correlates alongside care-related implications described in the literature.

Several limitations should also be acknowledged. First, the included studies were highly heterogeneous in design, setting, population, outcome definition, and ascertainment method, which limited comparability and precluded quantitative pooling. The strength and interpretation of these associations varied according to study design. Findings from larger epidemiological and longitudinal studies were considered more robust in interpretation, whereas observations derived from smaller cross-sectional or clinically selected samples were interpreted more cautiously. Second, prevalence estimates and care-related findings were not reported consistently across all studies. Third, definitions of co-occurrence varied substantially, with some studies reporting formal diagnoses, others reporting symptom-level findings, and others reporting broader psychosocial or developmental correlates. Fourth, the search strategy was primarily designed to identify epidemiological studies reporting prevalence patterns and associated correlates. As a result, literature focused primarily on service models may have been undercaptured. Fifth, interpretation of subgroup differences was limited because several populations of potential interest were inconsistently described or underreported across the included studies. Finally, the review was limited to English-language publications, which may restrict broader generalizability.

## Conclusion

Co-occurring mental health and substance-related conditions were frequently reported among adolescents and young adults in high-risk and service-engaged populations in North America. Trauma exposure, ACEs, and broader social adversity were commonly reported across the reviewed literature and were frequently described alongside these presentations. At the same time, direct evidence for evaluated, integrated, youth-specific care models remained limited. Taken together, these findings support the relevance of developmentally appropriate, integrated, and trauma-informed approaches to care and highlight the need for further research directly evaluating service delivery models for this population.

## Supplementary Information

Below is the link to the electronic supplementary material.

Supplementary Material 1

## Data Availability

All data generated or analysed during this study are included in this published article and its supplementary information files.
