# Supplementary material for: Substance use and co-occurring mental health conditions among adolescents and young adults in North America: a systematic review
Source: BMC Psychiatry. 2026 Sep 3;26:703. doi: 10.1186/s12888-026-08580-2 (PMC13543466; doi:10.1186/s12888-026-08580-2)
Supplement: Supplementary file 1 [file 12888_2026_8580_MOESM1_ESM.docx]

**Appendix**

**Appendix A: Literature Search Strategies**

**Search strategy for Ovid MEDLINE (2014- July 31 2024)**

| Row | Search term |
| --- | --- |
| 1 | substance-related disorders/ or alcohol-related disorders/ or alcoholism/ or amphetamine-related disorders/ or cocaine-related disorders/ or marijuana abuse/ or narcotic-related disorders/ or opioid-related disorders/ or heroin dependence/ or morphine dependence/ or opiate overdose/ or opium dependence/ or phencyclidine abuse/ or "tobacco use disorder"/ |
| 2 | ((drug* or substance* or alcohol or opi* or cocaine or amphetamine or cannabis or tobacco or stimulant*) adj4 disorder*).tw,kw,kf. |
| 3 | 1 or 2 |
| 4 | mental disorders/ or anxiety disorders/ or obsessive-compulsive disorder/ or panic disorder/ or phobic disorders/ or phobia, social/ or "disruptive, impulse control, and conduct disorders"/ or mood disorders/ or "bipolar and related disorders"/ or bipolar disorder/ or depressive disorder/ or depressive disorder, major/ or dysthymic disorder/ or seasonal affective disorder/ or cyclothymic disorder/ or exp personality disorders/ or "schizophrenia spectrum and other psychotic disorders"/ or psychotic disorders/ or schizophrenia/ or "trauma and stressor related disorders"/ or adjustment disorders/ or stress disorders, traumatic/ or stress disorders, post-traumatic/ or stress disorders, traumatic, acute/ |
| 5 | ((psychiatric or mental) adj2 (disorder* or diagnos*)).tw,kw,kf. |
| 6 | comorbidity/ |
| 7 | (comorbid* or co-occur* or cooccur*).mp. |
| 8 | concurrent.tw,kw,kf. |
| 9 | 4 or 5 or 6 or 7 or 8 |
| 10 | adolescent/ or young adult/ |
| 11 | (adolescen* or teen* or youth* or (highschool or high school or secondary school or secondary education) or (young adj (adult* or man or men or women or woman or person or persons or people or students))).tw,kw,kf. |
| 12 | 10 or 11 |
| 13 | 3 and 9 and 12 |
| 14 | prevalence/ |
| 15 | risk/ or risk factors/ or poverty/ or social support/ or Helplessness, Learned/ or personal satisfaction/ or depression/ or anxiety/ or social determinants of health.mp. |
| 16 | (prevalence or risk* or protective factor*).tw,kw,kf. |
| 17 | 14 and 15 and 16 |
| 18 | 13 and 17 |
| 19 | limit 18 to yr="2014 -Current" |

**Secondary search strategy for Ovid MEDLINE (2014- September 27 2024)**

| Row | Search term |
| --- | --- |
| 1 | exp Substance-Related Disorders/ |
| 2 | ((drug* or substance* or alcohol or opi* or cocaine or amphetamine or cannabis or tobacco or stimulant* or addiction* or depend* or use*) adj4 disorder*).tw,kf. |
| 3 | 1 or 2 |
| 4 | exp Mental Disorders/ |
| 5 | ((psychiatr* or mental) adj2 (disorder* or diagnos* or illness*)).tw,kf. |
| 6 | 4 or 5 |
| 7 | exp Comorbidity/ |
| 8 | (comorbid* or co-occur* or concur*).tw,kf. |
| 9 | 7 or 8 |
| 10 | adolescent/ or young adult/ |
| 11 | (adolescen* or teen* or youth* or high school or secondary school or secondary education).tw,kf. |
| 12 | (young adj2 (adult* or m#n or wom#n or person* or people* or student*)).tw,kf. |
| 13 | 10 or 11 or 12 |
| 14 | prevalence/ |
| 15 | prevalence*.tw,kf. |
| 16 | 14 or 15 |
| 17 | risk/ or risk factors/ |
| 18 | risk*.tw,kf. |
| 19 | 17 or 18 |
| 20 | 3 and 6 and 9 and 13 and 16 and 19 |
| 21 | limit 20 to yr="2014 -Current" |

**Search strategy for EMBASE (2014- September 27 2024)**

| Row | Search term |
| --- | --- |
| 1 | exp drug dependence/ |
| 2 | ((drug* or substance* or alcohol or opi* or cocaine or amphetamine or cannabis or tobacco or stimulant* or addiction* or depend* or use*) adj4 disorder*).tw,kf. |
| 3 | 1 or 2 |
| 4 | exp mental disease/ |
| 5 | ((psychiatr* or mental) adj2 (disorder* or diagnos* or illness*)).tw,kf. |
| 6 | 4 or 5 |
| 7 | exp comorbidity/ |
| 8 | (comorbid* or co-occur* or concur*).tw,kf. |
| 9 | 7 or 8 |
| 10 | adolescent/ |
| 11 | young adult/ |
| 12 | (adolescen* or teen* or youth* or high school or secondary school or secondary education).tw,kf. |
| 13 | (young adj2 (adult* or m#n or wom#n or person* or people* or student*)).tw,kf. |
| 14 | 10 or 11 or 12 or 13 |
| 15 | prevalence/ |
| 16 | prevalence*.tw,kf. |
| 17 | 15 or 16 |
| 18 | risk/ or risk factor/ |
| 19 | risk*.tw,kf. |
| 20 | 18 or 19 |
| 21 | 3 and 6 and 9 and 14 and 17 and 20 |
| 22 | limit 21 to yr="2014 -Current" |

**Search strategy for PsycINFO (2014- September 27 2024)**

| Row | Search term |
| --- | --- |
| 1 | DE "Substance Related and Addictive Disorders" OR DE "Addiction" OR DE "Substance Use Disorder" |
| 2 | TI ( (drug* or substance* or alcohol or opi* or cocaine or amphetamine or cannabis or tobacco or stimulant* or addiction* or depend* or use*) N4 disorder* ) OR AB ( (drug* or substance* or alcohol or opi* or cocaine or amphetamine or cannabis or tobacco or stimulant* or addiction* or depend* or use*) N4 disorder* ) |
| 3 | S1 OR S2 |
| 4 | DE "Mental Disorders" OR DE "Affective Disorders" OR DE "Anxiety Disorders" OR DE "Behavior Disorders" OR DE "Bipolar Disorder" OR DE "Borderline States" OR DE "Chronic Mental Illness" OR DE "Dissociative Disorders" OR DE "Eating Disorders" OR DE "Gender Dysphoria" OR DE "Mental Disorders due to General Medical Conditions" OR DE "Neurocognitive Disorders" OR DE "Neurodevelopmental Disorders" OR DE "Neurosis" OR DE "Obsessive Compulsive Disorder" OR DE "Paraphilias" OR DE "Personality Disorders" OR DE "Psychosis" OR DE "Serious Mental Illness" OR DE "Sleep Wake Disorders" OR DE "Somatoform Disorders" OR DE "Stress and Trauma Related Disorders" OR DE "Substance Related and Addictive Disorders" OR DE "Thought Disorders" |
| 5 | TI ( (psychiatr* or mental) N2 (disorder* or diagnos* or illness*) ) OR AB ( (psychiatr* or mental) N2 (disorder* or diagnos* or illness*) ) |
| 6 | S4 OR S5 |
| 7 | DE "Comorbidity" |
| 8 | TI ( comorbid* or co-occur* or coocur* or concur* ) OR AB ( comorbid* or co-occur* or coocur* or concur* ) |
| 9 | S7 OR S8 |
| 10 | DE "Late Adolescence" or DE "Early Adolescence" |
| 11 | TI ( adolescen* or teen* or youth* or high school or secondary school or secondary education ) OR AB ( adolescen* or teen* or youth* or high school or secondary school or secondary education ) |
| 12 | TI ( young N2 (adult* or m#n or wom#n or person* or people* or student*) ) OR AB ( young N2 (adult* or m#n or wom#n or person* or people* or student*) ) |
| 13 | S10 OR S11 OR S12 |
| 14 | DE "Morbidity" |
| 15 | TI prevalence* OR AB prevalence* |
| 16 | S14 OR S15 |
| 17 | DE "Risk Factors" OR DE "At Risk Populations" |
| 18 | TI risk* OR AB risk* |
| 19 | S17 OR S18 |
| 20 | S3 AND S6 AND S9 AND S13 AND S16 AND S19 |

**Grey literature search (Google Scholar)**

Google Scholar was searched using an adapted keyword combination based on the core review concepts of adolescents and young adults, substance use or substance-related problems, and co-occurring mental health conditions. Citations, review articles, and studies conducted outside North America were not included. The first 150 results were screened; 142 records with a relevant topic and of an accepted type were retrieved and entered into the review.

**Appendix B: Results of Risk of Bias Assessment**

**Supplementary Table 1A:** Results of the risk of bias assessment for included cross-sectional studies. Risk of bias was assessed using the JBI critical appraisal checklist (2025 version) for analytical cross-sectional studies. The revised checklist of cross-sectional studies (electronically published in May 2025; subsequently published in the 2026 journal issue) was available through JBI at the time of access. The two critical domains identified for risk of bias assessment were confounding factors (assessed by Q5, Q6), and measurement validity and reliability (assessed by Q3, Q7).

Green (🟢) = (Yes), Red (🔴) = (No), Yellow (🟡) = (Unclear)

| Study | Q1 | Q2 | Q3 | Q4 | Q5 | Q6 | Q7 | Q8 |
| --- | --- | --- | --- | --- | --- | --- | --- | --- |
| Adams et al., 2015 | 🟢 | 🟢 | 🟢 | 🟢 | 🟢 | 🟢 | 🟢 | 🟢 |
| Cheung et al., 2019 | 🟢 | 🟢 | 🟢 | 🟢 | 🟢 | 🟢 | 🟢 | 🟢 |
| Brinkman et al., 2015 | 🟢 | 🟢 | 🟢 | 🟢 | 🟢 | 🟢 | 🟢 | 🟢 |
| Brownlie et al., 2019 | 🟢 | 🟢 | 🟢 | 🟢 | 🟢 | 🟢 | 🟢 | 🟢 |
| Carliner et al., 2017 | 🟢 | 🟢 | 🟢 | 🟢 | 🟢 | 🟢 | 🟢 | 🟢 |
| Conway et al., 2016 | 🟢 | 🟢 | 🟢 | 🟢 | 🟢 | 🟢 | 🟢 | 🟢 |
| Masroor et al., 2019 | 🟢 | 🟢 | 🟢 | 🟢 | 🟢 | 🟢 | 🟢 | 🟢 |
| Edlund et al., 2015 | 🟢 | 🟢 | 🟢 | 🟢 | 🟢 | 🟢 | 🟢 | 🟢 |
| Freibott et al., 2024 | 🟢 | 🟢 | 🟢 | 🟢 | 🟢 | 🟢 | 🟢 | 🟢 |
| Goodwin et al., 2014 | 🟢 | 🟢 | 🟢 | 🟢 | 🟢 | 🟢 | 🟢 | 🟢 |
| Hawke et al., 2018 | 🟢 | 🟢 | 🟢 | 🟢 | 🟡 | 🟡 | 🟢 | 🟢 |
| Henderson et al., 2017 | 🟢 | 🟢 | 🟢 | 🟢 | 🟡 | 🟡 | 🟢 | 🟢 |
| Leonard et al., 2023 | 🟢 | 🟢 | 🟢 | 🟢 | 🟢 | 🟢 | 🟢 | 🟢 |
| Mann et al., 2014 | 🟢 | 🟢 | 🟢 | 🟢 | 🟢 | 🟢 | 🟢 | 🟢 |
| McKnight et al., 2023 | 🟢 | 🟢 | 🟢 | 🟢 | 🟢 | 🟢 | 🟢 | 🟢 |
| Regan & Tubman, 2020 | 🟢 | 🟢 | 🟢 | 🟢 | 🟢 | 🟢 | 🟢 | 🟢 |
| Salmon et al., 2023 | 🟢 | 🟢 | 🟢 | 🟢 | 🟢 | 🟢 | 🟢 | 🟢 |
| Single et al., 2024 | 🟢 | 🟢 | 🟢 | 🟢 | 🟢 | 🟢 | 🟢 | 🟢 |
| Sultan et al., 2025 | 🟢 | 🟢 | 🟢 | 🟢 | 🟢 | 🟢 | 🟢 | 🟢 |
| Ware et al., 2024 | 🟢 | 🟢 | 🟢 | 🟢 | 🟢 | 🟢 | 🟢 | 🟢 |
| Xu et al., 2024 | 🟢 | 🟢 | 🟢 | 🟢 | 🟢 | 🟢 | 🟢 | 🟢 |
| Aderibigbe et al., 2022 | 🟢 | 🟢 | 🟢 | 🟢 | 🟢 | 🟢 | 🟢 | 🟢 |
| Desai et al., 2022 | 🟢 | 🟢 | 🟢 | 🟢 | 🟢 | 🟢 | 🟢 | 🟢 |
| Striley et al., 2022 | 🟢 | 🟢 | 🟢 | 🟡 | 🟢 | 🟢 | 🟢 | 🟢 |
| Dawson-Rose et al., 2020 | 🟢 | 🟢 | 🟢 | 🟢 | 🔴 | 🔴 | 🟢 | 🟢 |

**Supplementary Table 1B:** Results of the risk of bias assessment for included cohort studies. Risk of bias was assessed using the JBI critical appraisal checklist (2025 version) for cohort studies. The revised checklist of cohort studies (electronically published in March 2025) was available through JBI at the time of access. The four critical domains identified for risk of bias assessment were confounding factors (assessed by Q1, Q4, Q5), exposure (assessed by Q6), measurement validity and reliability (assessed by Q3, Q7), and attrition and follow up (assessed by Q8, Q9).

Green (🟢) = (Yes), Red (🔴) =(No), Yellow (🟡) = (Unclear), Blue (🔵) = (Not Applicable)

| Study | Q1 | Q2 | Q3 | Q4 | Q5 | Q6 | Q7 | Q8 | Q9 | Q10 | Q11 |
| --- | --- | --- | --- | --- | --- | --- | --- | --- | --- | --- | --- |
| Krebs et al., 2021 | 🟢 | 🟢 | 🟢 | 🟢 | 🟢 | 🟢 | 🟢 | 🔵 | 🔵 | 🔵 | 🟢 |
| Ramos-Olazagasti et al., 2017 | 🟢 | 🟢 | 🟢 | 🟢 | 🟢 | 🟡 | 🟢 | 🟢 | 🟢 | 🟡 | 🟢 |
| Rhew et al., 2017 | 🟢 | 🟢 | 🟢 | 🟢 | 🟢 | 🟡 | 🟢 | 🟢 | 🟢 | 🟢 | 🟢 |

**Supplementary Table 1C:** Results of the risk of bias assessment for included case control studies. Risk of bias was assessed using the JBI critical appraisal checklist for case control studies (2020 version). The checklist for case control studies published in 2020 is the latest available version accessible through the JBI website. The three critical domains identified for risk of bias assessment were those to do with comparability and matching between groups (assessed by Q1, Q2), measurement validity and reliability (assessed by Q4,Q5), and confounding factors (assessed by Q6, Q7).

Green (🟢) = (Yes), Red (🔴) = (No), Yellow (🟡) = (Unclear)

| Study | Q1 | Q2 | Q3 | Q4 | Q5 | Q6 | Q7 | Q8 | Q9 | Q10 |
| --- | --- | --- | --- | --- | --- | --- | --- | --- | --- | --- |
| Marshall et al., 2023 | 🟢 | 🟢 | 🟢 | 🟢 | 🟢 | 🟢 | 🟢 | 🟢 | 🟢 | 🟢 |

**Appendix C: PRISMA flow diagram**

**Appendix D**

**Supplementary Table 2:** Additional details of studies, including aim, instruments for identification, any reported prevalence of concurrent disorders or symptoms, and associated/contextual domains.

| **Study** | **Aim** | **Instruments for Identification** | **Prevalence of Concurrent Disorders or Symptoms** | **Associated/ Contextual Domains** |
| --- | --- | --- | --- | --- |
| Leonard et al., 2023 | To examine factors associated with methamphetamine use among TAY | Survey, includes:  Center for Epidemiologic Studies Depression Scale (CES-D): Symptom severity measure correlated with DSM-defined depression  PTSD Checklist for DSM-5 (PCL-5): Symptom-based assessment aligned with  DSM-5 PTSD criteria  Generalized Anxiety Disorder 7-item Scale (GAD-7): Symptom severity measure correlated with DSM-defined anxiety disorders  Adverse Childhood Experiences (ACEs) Questionnaire: Exposure-based assessment of childhood trauma (risk factor measure) | Meth use and:  Depression - 89.3% (vs. 67.1% non-users)  PTSD - 96.4% (vs. 72.7% non-users)  Moderate/Severe Anxiety - 57.1% (vs. 47.1% non-users) | N/A |
| Mann et al.,  2014 | To examine prevalence and risk factors associated with SU in adolescents with EDs | Eating Disorder Examination (EDE): Symptom-based assessment that can generate DSM diagnoses  Schedule for Affective Disorder and Schizophrenia for School-Age Children (K-SADS): DSM-based diagnostic interview  Beck Depression Inventory (BDI): Symptom severity measure correlated with DSM-defined depression | Lifetime SU (any history of SU, even single instances) and:  BN -  48.7%  Eating Disorder Not Otherwise Specified (EDNOS) -  28.6%  AN -  24.6%  Regular Use or SUD (more severe, patterned use):  Overall prevalence -  27.9%  BN - 32.4%  EDNOS - 20.7%  AN - 16.1% | Male Gender  Non-White Race (racial/ethnic minority) |
| Rhew et al.,  2017 | To estimate the effects of cumulative depression during early adolescences and the likelihood of CUD and AUD at age 18 | Developmental Pathways Project (DPP) data, includes:  Diagnostic Interview Schedule for Children (DISC-IV) Depression Module: DSM-based diagnostic interview for depressive disorders  Voice Diagnostic Interview Schedule for Children (V-DISC): DSM-based diagnostic interview, two modules selected for substance use disorders (including cannabis use disorder and alcohol use disorder) | Depression and:  CUD - 20.9%  AUD - 19.8% | N/A |
| Adams et al.,  2015 | To estimate the prevalence and risk factors for comorbid PTSD, MDE, and SUD | Structured interview, includes:  National Survey of Adolescents PTSD Module (NSA-PTSD): DSM-based diagnostic interview for post-traumatic stress disorder (PTSD)  National Survey of Adolescents Depression Module (NSA-Depression): DSM-based diagnostic interview for major depressive episode (MDE)  CRAFFT Screening Test: Symptom self-report screening tool for substance use disorders (SUD) | SUD and:  PTSD - 1.1%  MDE - 1.0%  PTSD and MDE - 0.7% | Prior Traumatic Events  Female Gender  Loss of Services (e.g., utilities, shelter)  Non-White Race |
| Goodwin et al.,  2014 | To investigate hookah use prevalence, demographic correlates, associations with SU, MH, and stress | American Health Association-National College Health Assessment (ACHA-NCHA), includes items on substance use, mental health, and stress. | Hookah use and:  Major depression -  7.6%  Anxiety disorders - 8.3% | N/A |
| Sultan et al.,  2025* | To examine prevalence and correlates of CU and CUD among adolescents with MDD or BD | National Comorbidity Survey–Adolescent Supplement (NCS-A), includes:  Modified version of Composite International Diagnostic Interview (CIDI): DSM-IV–based diagnostic interview generating diagnoses for mood disorders (major depression, dysthymia, bipolar I/II), anxiety disorders, eating disorders, behavior disorders (oppositional defiant disorder and conduct disorder), attention-deficit/hyperactivity disorder (ADHD), and substance use disorders.  Cannabis use/cannabis use disorder items: DSM-IV–based assessment of cannabis abuse and dependence, including symptom criteria, patterns of use, and age of onset. | **MDD:**  CU -  32.9%  CUD - 8.0%  **BD:**  CU - 25.6%  CUD - 2.6%  **Controls:**  CU - 16.9%  CUD - 3.7%  **AUD Comorbidity with:**  MDD and  CUD - 37.4%  BD and CUD - 29.8% | Lifetime suicidal ideation/attempts  AUD  Treatment Seeking for Depression  Lifetime CD  Physical/sexual abuse history  Longer depressive episodes  Female sex |
| Freibott et al.,  2024 | To estimate the prevalence of opioid misuse, examine correlates with anxiety and depression symptoms, and quantify help-seeking behaviors | Healthy Minds Study (HMS), includes:  Patient Health Questionnaire-9 (PHQ-9): Symptom self-report measure aligned with DSM-defined depression.  Generalized Anxiety Disorder-7 (GAD-7): Symptom self-report measure aligned with DSM-defined generalized anxiety disorders  Self-reported mental health diagnosis item: indicator of clinician-diagnosed mental health conditions  Self-report substance use item (past 30-day opioid misuse) | Opioid misuse and:  Any MH diagnosis - 57.9%  Anxiety/depression - 64.3% | N/A |
| Single et al.,  2024 | To examine the association between CU and SAD in emerging adulthood and identify sociodemographic and psychiatric disorder correlates | National Epidemiologic Survey on Alcohol and Related Conditions–III (NESARC-III), includes:  Alcohol Use Disorder and Associated Disabilities Interview Schedule-5 (AUDADIS-5): DSM-5–based structured diagnostic interview assessing cannabis use, social anxiety disorder, and other psychiatric disorders, including major depressive disorder, bipolar I disorder, generalized anxiety disorder, specific phobia, agoraphobia, and panic disorder. | Lifetime CU and past-year SAD - 1.10% | White race/ethnicity  Part-time or non-student status  Comorbid psychiatric disorders (MDD, Bipolar I, GAD, Specific Phobia, Agoraphobia, Panic Disorder) |
| Xu et al.,  2024 | To examine the association of adolescent SU and depressive symptoms with adult MDD | National Survey on Drug Use and Health (NSDUH), includes:  Major Depressive Episode (MDE) items: DSM-IV/DSM-5–based assessment of major depressive disorder (MDD) symptoms and diagnosis.  Substance Use Disorder (SUD) items: DSM-based assessment of illicit drug and alcohol abuse and dependence. | SUD and MDD -  20.5% | Female  Young age (18-25)  Low-income individuals (earning under $50K/year) |
| McKnight et al.  2023 | To compare standardized measures of ACEs and protective factors among AYAs | Formal diagnosis based on DSM for opioid use disorder (OUD) and feeding and eating disorders (FEDs) | No. of ACEs endorsed by AYA with OUDs and FEDs:  0: 17 (18.9%)  1: 9 (10%)  2: 7 (7.8%)  3: 8 (8.9%)  4: 49 (54.4%)  ≥4 ACEs reported by 80.0% of participants with OUD versus 34.0% with FEDs | Higher ACE scores (score of ≥4)  Lower resilience factors |
| Striley et al.,  2022 | To evaluate the relationship between the use of e-cigarettes, marijuana use, smoking, and alcohol use, and non-suicidal self-injury (NSSI) behavior | Healthy Minds Study (HMS), includes:  Non-Suicidal Self-Injury (NSSI) Behavior Items: Self-report measure of non-suicidal self-injurious behaviors and frequency  E-cigarette Use Items: Self-report measure of current e-cigarette/vaping use and substance vaped  Conventional Cigarette Use Item: Self-report measure of current cigarette use  Other Substance Use Items: Self-report measure of past-30-day use of marijuana and other substances  Patient Health Questionnaire-9 (PHQ-9): Symptom self-report measure aligned with DSM-defined depression  Generalized Anxiety Disorder-7 (GAD-7): Symptom self-report measure aligned with DSM-defined anxiety disorders | Of those with past-year self-injury:  Electronic cigarette use = 27%  Conventional cigarette use = 16.2%  Marijuana use = 37.6%  Alcohol use = 61.5%  Use of other substances = 9.0% | Female  White  Experience heightened symptoms of depression or anxiety  Engagement in vaping, smoking, and  other drug use |
| Salmon et al.,  2023 | To understand the patterns of exposure to several types of child maltreatment and peer victimization among  adolescents | Ontario Child Health Study, includes:  Self-reported substance use items assessing recent smoking, alcohol use, cannabis use, illicit drug use, and non-medical prescription drug use. | N/A | Child maltreatment  (physical abuse, emotional abuse, sexual abuse, physical neglect, exposure to intimate partner violence )  Peer Victimization (physical, verbal, social, cyber, discrimination) |
| Krebs et al.,  2021 | To describe trends in the number of youths diagnosed with OUD and to identify  factors associated with OUD diagnosis in acute care settings | Administrative health databases:  OUD: ICD-9/ICD-10 diagnosis codes, Drug Identification Numbers (DINs), Product Identification Numbers (PINs) for opioid agonist treatment  Comorbid conditions at the time of OUD diagnosis: ICD-9/ ICD-10 diagnosis codes in physician billing and hospitalization records | (By group)  OUD with MH Disorder:  Adolescents - 79.2%  Young Adults - 68% | N/A |
| Dawson-Rose et al.,  2020 | To describe trauma, mental health symptoms, and substance use in TAY who are experiencing homelessness or at risk of becoming homeless | Survey, includes:  PTSD Checklist for DSM-5 (PCL-5): Self-report measure of PTSD symptom severity, used for screening and monitoring symptom intensity  Center for Epidemiologic Studies Depression Scale (CES-D): Self-report measure of depressive symptoms and risk for clinical depression  Generalized Anxiety Disorder-7 (GAD-7): Self-report measure of anxiety symptoms and severity  NIDA Quick Screen V1.0: Brief self-report screening tool identifying past-year binge drinking and use of tobacco, illicit drugs, or non-medical prescription drugs  NIDA-Modified Alcohol, Smoking, and Substance Involvement Screening Test (ASSIST) V2.0: Self-report measure assessing past-3-month substance use and level of risk for harmful substance use across multiple substances | N/A | For SU:  Exposure to abuse, neglect, or household dysfunction during childhood  PTSD, anxiety, and depression symptoms |
| Regan & Tubman, 2020 | To examine the prevalence of ADHD subtypes among adolescents receiving SU treatment | Brief Michigan Version of the Composite International Diagnostic Interview (UM-CIDI): DSM-IV–based structured diagnostic interview administered by trained interviewers to assess past-year psychiatric disorders, including ADHD subtypes, conduct disorder/oppositional defiant disorder, affective disorders (major depressive disorder, dysthymia), anxiety disorders (generalized anxiety disorder, specific phobia, social phobia, panic disorder), and alcohol and drug abuse/dependence disorders | N/A | ADHD-Inattentive subtype, ADHD Hyperactive-Impulsive, ADHD Combined subtype diagnoses  Female/girls |
| Cheung et al., 2019 | To examine co-occurring MH problems among the sample to understand prevalence and associated risk factors | Ontario Student Drug Use and Health Survey (OSDUHS), includes:  Kessler 6-item Psychological Distress Scale (K6): Measures symptoms of anxiety and depression; used to assess psychological distress in the past 12 months.  Suicide Ideation and Suicide Attempt Items: Self-report items assessing whether students seriously considered attempting suicide or attempted suicide in the past 12 months.  Alcohol Use Disorders Identification Test (AUDIT): Assesses hazardous and harmful drinking.  CRAFFT Screener: Assesses drug use problems experienced in the past month.  Severity of Dependence Scale (SDS): Screens for cannabis dependence. | Overall co-occurring problems*: 12.0% (10.0–14.3%)  *SU problems (cannabis dependence  drug use problem, hazardous/harmful drinking) and internalizing symptoms (elevated psychological distress suicide ideation, suicide attempt, and overall internalizing symptoms) | N/A |
| Ramos-Olazagasti et al., 2017 | To examine association between childhood adversity and early initiation of alcohol use | Diagnostic Interview Schedule for Children, Version IV (DISC-IV) – Alcohol Abuse Module: DSM-based structured diagnostic interview  Alcohol Use Initiation Items: Self-report items assessing lifetime alcohol use and age at first use | N/A | Accumulation of childhood adversities physical abuse, emotional abuse, parental antisocial personality, and exposure to violence  exposure to violence (In girls)  Early alcohol use |
| Masroor et al., 2019 | To compare demographics of CD in the sample and examine association with SUD | Nationwide Inpatient Sample (NIS) database (HCUP), includes:  Conduct disorder diagnoses: Identified using ICD-9 diagnostic codes in principal diagnosis fields  Substance Use Disorders (tobacco, cannabis, opioid, cocaine, amphetamine, alcohol): Identified using ICD-9 diagnostic codes in secondary diagnosis fields | CD and:  AU - 10.1%  Tobacco use -  10.1%  CU - 23.7%  Opioid use - 1.6%  Cocaine/Amphetamine use - 2.8% | CD - higher odds for CUD and SUD  Male gender |
| Conway et al., 2016 | To estimate the association of prior lifetime mental disorders with transitions across stages of substance use | National Comorbidity Survey–Adolescent Supplement (NCS-A), includes:  Composite International Diagnostic Interview (CIDI) modified version: DSM-IV–based structured diagnostic interview generating diagnoses for mood disorders, anxiety disorders, behavior disorders, eating disorders, and lifetime substance use disorders (alcohol and drug abuse/dependence)  Substance Use and Age-of-Onset Items: Self-report measures assessing lifetime use of alcohol and illicit drugs, age of first use, first regular use, and first opportunity to use substances. | **Alcohol abuse with any:**  Mood disorder -  13.9%  Phobia disorder - 5%  Anxiety disorder - 17.3%  Behavior disorder - 15.6%  Eating disorder - 7%  **Illicit drug abuse with any:**  Mood disorder -  19.3%  Phobia disorder - 12%  Anxiety disorder - 20%  Behavior disorder - 24%  Eating disorder - 18.4% | Prior mental disorder -  Increased likelihood of progressing through stages of alcohol and drug use  Prior SU - Increased risk of alcohol use progression  behavioral and mood disorder - Alcohol and SU progression |
| Edlund et al., 2015 | To investigate the association of MDE with nonmedical prescription opioid use (NMPOU) and opioid abuse/ dependence | National Survey on Drug Use and Health (NSDUH), includes:  Nonmedical Prescription Opioid Use (NMPOU) item: Self-report measure of opioid misuse  Opioid Abuse/Dependence: DSM-IV–based assessment of opioid use disorder  Major Depressive Episode (MDE) questionnaire (based on NCS-A depression module): DSM-based assessment of depression  Alcohol Use Disorder: DSM-IV–based assessment of alcohol abuse/dependence  Nonopioid Drug Abuse/Dependence: DSM-IV–based assessment of substance use disorders (e.g., marijuana, cocaine, stimulants, hallucinogens, inhalants, tranquilizers, sedatives) | Past-year MDE with past-year NMPOU - 20%  NMPOU with opioid abuse/dependence - 15% | Female  Had a nonopioid drug use disorder and AUD  Less family support  More delinquent behaviors  A greater frequency of religious service attendance  Adolescents who had a greater likelihood of opioid abuse or dependence |
| Desai et al., 2022 | To evaluate the prevalence and odds of hopelessness, suicidality, and co-occurring substance use among adolescents using hallucinogens | Centers for Disease Control and Prevention’s Youth Risk Behavior Surveillance System (YRBSS) survey, includes:  Hallucinogen Use Item: Self-report measure of lifetime hallucinogen use  Co-occurring Substance Use Items: Self-report measures of lifetime use of cigarettes, e-cigarettes, synthetic marijuana, cocaine, inhalants, heroin, methamphetamine, ecstasy, and age at first alcohol use  Mental Health Items: Self-report measures of sadness/hopelessness and suicidality (suicide ideation, plan, attempts, and injurious attempts) | Ever hallucinogen use and:  Injurious suicide attempts - 12%  Attempted suicide - 24.6%  Made a suicide plan - 32.6%  Considered suicide - 36.3%  Sad and hopeless - 48.4% | Hallucinogen use  - associated with feeling sad and hopeless, considering suicide, and planning suicide, SU |
| Carliner et al., 2017 | To examine associations of potentially traumatic events (PTEs) with ODD, CU, and SUD | National Comorbidity Survey Replication–Adolescent Supplement (NCS-A), includes:  Composite International Diagnostic Interview (CIDI): DSM-IV–based diagnostic interview assessing externalizing disorders  Self-Administered Questionnaire (SAQ) – Parent Report: Parent-reported assessment of child externalizing psychopathology  Oppositional Defiant Disorder (ODD) and Conduct Disorder (CD): DSM-IV–based diagnoses using combined parent and adolescent reports  Substance Use Disorders (SUDs): DSM-IV–based diagnoses (adolescent report only) for alcohol abuse/dependence, drug abuse/dependence, and nicotine dependence | **SUD and:**   Any PTE exposure - 18.91%  Interpersonal trauma exposure - 31.20%  Accidental trauma exposure - 19.80%  Network/witnessing trauma exposure - 20.99%  Other/Non-disclosed trauma exposure - 31.15% | For SUD and CD: Exposure to interpersonal violence  Exposure to accidental/nonintentional events, network/witnessing events and other events |
| Brinkman et al., 2015 | To evaluate the association of ADHD and CD with early tobacco and alcohol use | National Health and Nutrition Examination Survey (NHANES), includes:  Diagnostic Interview Schedule for Children (DISC): DSM-IV–based structured diagnostic interview assessing ADHD and conduct disorder (CD) diagnoses and symptom counts  Audio Computer Assisted Self-Interview (A-CASI): Self-report assessment of lifetime tobacco and alcohol use and age of initiation (behavioral self-report) | **Alcohol use with:**  ADHD and CD - 54.2%  ADHD only - 27.8%  CD only -  29.1%  **Tobacco use with:**  ADHD and CD -  48.2%  ADHD only - 38.1%  CD only - 59.9% | ADHD and CD diagnoses - increased likelihood of SU, using tobacco and alcohol, and initiated use at a younger age |
| Henderson et al., 2017 | To explore characteristics of NEET youth compared to non-NEET peers | Global Screener (GSS)- Extended Version (adapted from the GAIN-I): Self-report screening tool assessing likelihood of internalizing disorders, externalizing disorders, substance use disorders, crime/violence issues, and additional domains (eating-related issues, trauma, thought disturbance, gaming/internet overuse, gambling). Identifies probable need for services rather than DSM diagnoses. | **Non-NEET:**  Internalizing disorder with SUD - 5.1%  Externalizing disorder with SUD - 3.5%  Internalizing and externalizing disorder with SUD - 24.4%  **NEET:**  Internalizing disorder with SUD - 10.7%  Externalizing disorder with SUD - 4.5%  Internalizing and externalizing disorder with SUD - 33.3% | NEET - associated with problematic SU, crime/violence, disturbing memories |
| Hawke et al., 2018 | To describe the rates of CU, SU profiles, and MH characteristics | Adolescent Alcohol and Drug Involvement Scale (AADIS): Self-report measure of lifetime and frequency of substance use and age of first use  Target Substance Item: Self-report item identifying substances of greatest concern or desired change  Global Appraisal of Individual Needs- Short Screener (GAIN-SS): Self-report screening tool identifying likelihood of internalizing disorders, externalizing disorders, and substance use disorders. | **Male:**  Internalizing disorder with SUD - 15.1%  Externalizing disorder with SUD - 8.7%  Internalizing and externalizing disorder with SUD - 52.4%  **Female:**  Internalizing disorder with SUD - 15%  Externalizing disorder with SUD - 4.7%  Internalizing and externalizing disorder with SUD - 71.7%  **Adolescents:**  Internalizing disorder with SUD - 6.2%  Externalizing disorder with SUD - 12%  Internalizing and externalizing disorder with SUD - 59.5%  **Young adults:**  Internalizing disorder with SUD -  17.5%  Externalizing disorder with SUD - 4.3%  Internalizing and externalizing disorder with SUD - 59.6% | Female - more likely to have co-occurring MH problems    Adolescents - more likely to have co-occurring externalizing disorders and SUD    Young adults - more likely to have co-occurring internalizing disorders and SUD |
| Brownlie et al., 2019 | To examine the overlap between MH problems and SU | Global Appraisal of Individual Needs- Short Screener (GAIN-SS): Self-report screening tool identifying likelihood of internalizing disorders, externalizing disorders, and substance use disorders  Adapted Ontario Student Drug Use and Health Survey (OSDUHS) Survey Items: Self-report measures of demographics, substance use (alcohol and other substances), and mental health service utilization | Concurrent mental health and substance use problem - 5.73% | N/A |
| Marshall et al., 2023 | To explore whether the onset of OUD is associated with preexisting MH conditions such as anxiety and depressive disorders in young people | Administrative health databases:  ICD-based identification of OUD diagnoses and enrollment in an opioid dependence program  Mental Health Diagnoses: ICD-based identification of anxiety disorders, depressive disorders, co-occurring anxiety and depressive disorders, alcohol-related disorders, and substance-related disorders (recorded prior to OUD diagnosis) | **OUD with:**  Anxiety disorders - 72.7%  Depressive disorders - 34.7%  Alcohol-related disorders - 29.0%  Anxiety and depressive disorders - 31.8%  Anxiety and alcohol-related disorders - 25.3%  Depressive and alcohol-related disorders - 17.2%  Anxiety, depressive, and alcohol-related disorders - 16.2% | For OUD - Preexisting and/or co-occurring anxiety, depressive, and alcohol-related disorders |
| Ware et al., 2024 | To identify the proportion of youth with anxiety disorders, depressive disorders, or ADHD as a primary diagnosis | Mental Health Client-Level Data, contains annual cross-sectional administrative data from mental health treatment facilities. includes:  Mental Health Diagnosis Variables: Administrative/record-based indicators of diagnosed conditions (e.g., anxiety, ADHD, conduct disorder, depressive disorder, oppositional defiant disorder, trauma- and stressor-related disorders, and other mental health disorders)  Substance Use Disorder (SUD) / Substance Use Problem Variable: Composite administrative measure combining diagnosis and other identification methods (e.g., screening results, program enrollment, survey, or service records) to indicate high-risk substance use or SUD | High-risk SU or SUD with:  Anxiety -  5.2%  Depression - 9.8%  ADHD - 5.2% | For SU/SUD with either anxiety, depression, or ADHD:  Older youth  Male/boys  Another race or ethnicity other than White  Co-occurring MH conditions (CD, ODD, trauma/stressor-related disorders, and other MH disorders) |
| Aderibigbe et al., 2022 | To examine individual factors associated with SU | interRAI Child and Youth Mental Health Assessment (ChYMH), includes:  Anxiety Scale: Symptom self-report measure of anxiety severity  Hyperactive/Distraction Scale: Symptom self-report measure of distractibility and hyperactivity  Depression Severity Index: Symptom self-report measure of depressive symptoms  Substance Use Items: Self-report measure of recent substance use and medication misuse | N/A | Older age  Self-injurious ideation/attempt  behavior symptoms  Problematic sexual behavior  Being a victim of abuse  Being at risk of disrupted education  Lack of family support  Having parent/caregiver with SUD |

*Available online at the time of the search; Formally published in 2025

ACE: adverse childhood experience; ADHD: attention deficit hyperactivity disorder; AN: anorexia nervosa; AUD: alcohol use disorder; AUDIT: Alcohol Use Disorder Identification Test; AYA: adolescents and young adults; BD: bipolar disorder; BN: bulimia nervosa; CD: conduct disorder; CIDI: Composite International Diagnostic Interview; CU: cannabis use; CUD: cannabis use disorder; ED: eating disorder; GAD: generalized anxiety disorder; GAD-7: Generalized Anxiety Disorder-7 Questionnaire; GAIN-SS: Global Appraisal of Individual Needs-Short Screener; MDD: major depressive disorder; MDE: major depressive episode; MH: mental health; NEET: Not in employment, education, or training; NMPOU: nonmedical prescription opioid use; NSSI: non-suicidal self-injury; ODD: oppositional defiant disorder; OUD: opioid use disorder; PTSD: post-traumatic stress disorder; SAD: social anxiety disorder; SUD: substance use disorder; TAY: transitional aged youth; N/A: not available
